# Cellular Architecture of Spinal Granulomas and the Immunological Response in Tuberculosis Patients Coinfected with HIV

**DOI:** 10.3389/fimmu.2017.01120

**Published:** 2017-09-11

**Authors:** Debapriya Bhattacharya, Siva Danaviah, Daniel M. Muema, Ngomu Akeem Akilimali, Prashini Moodley, Thumbi Ndung’u, Gobardhan Das

**Affiliations:** ^1^Special Centre for Molecular Medicine (SCMM), Jawaharlal Nehru University, New Delhi, India; ^2^Medical Microbiology Laboratory, School of Health Sciences, University of KwaZulu-Natal, Durban, South Africa; ^3^Africa Health Research Institute, Durban, South Africa; ^4^HIV Pathogenesis Programme, Doris Duke Medical Research Institute, University of KwaZulu-Natal, Durban, South Africa; ^5^Max Planck Institute for Infection Biology, Berlin, Germany; ^6^Ragon Institute of Massachusetts General Hospital, Massachusetts Institute of Technology, Harvard University, Cambridge, MA, United States

**Keywords:** tuberculosis, HIV, extrapulmonary tuberculosis, T cell, B cell, granuloma, mesenchymal stem cells

## Abstract

*Mycobacterium tuberculosis* (*M.tb*) and HIV are individually responsible for the most deaths worldwide among all infectious agents, and coinfection with *M.tb* and HIV is a significant public health challenge in the developing world. Although the lung is the primary target organ for tuberculosis (TB), *M.tb* can also cause extrapulmonary tuberculosis (EPTB) such as in the bones and joints. Treatment of EPTB is much more challenging than treatment of pulmonary TB. The hallmark of the host immune response against TB is the formation of organized structures called granulomas that are infiltrated with immune cells and are rich in cytokines and chemokines. Inside granulomas, the host confines the *M.tb* bacteria to a particular region of the organ and avoids dispersion. In this study, we analyzed immune cells in bone granulomas of patients with EPTB that are also coinfected with HIV. We found that HIV-infected TB patients have dispersed bone granulomas, with reduced T cell numbers and a concomitant increase in plasma cells. Additionally, HIV-infected patients exhibited dramatically increased serum levels of IgM and IgG1 antibodies, which is indicative of T-cell-independent B-cell activation and mucosal T-cell activation, respectively. Interestingly, we also observed that CD29^+^ stem cells are increased in HIV–TB coinfection, suggesting a link with HIV infection. Therefore, our work provides new insights into the architecture of spinal TB granulomas and the role of B-cells and humoral immunity against a highly infectious intracellular pathogen. We propose that our findings will inform biomarker identification for EPTB and possibly the development of related therapeutics and/or vaccines to protect HIV-infected patients against disseminated TB.

## Introduction

Despite recent advances in diagnosis and treatment, *Mycobacterium tuberculosis* (*M.tb*) poses a significant global public health threat (1). Only 10% of all individuals exposed to this pathogen develop active disease immediately and the remaining individuals either confine or eradicate the pathogen, depending on their immune status. The hallmark of the host immune response against tuberculosis (TB) is the formation of an organized structure called a granuloma that is infiltrated with immune cells and is rich in cytokines and chemokines (1). Immune compromised hosts, however, are unable to mount an effective immune response and, therefore, develop active disease, progressing rapidly to severe morbidity and even mortality.

The most common cause of immune perturbation in *M.tb*-infected individuals is HIV coinfection (2). Both TB and HIV have profound effects on the immune system, as they are capable of disarming the host’s immune defenses through mechanisms that are not fully understood. HIV coinfection is the most significant known risk factor for progression of *M.tb* infection to active disease, increasing the risk of reactivation of latent TB by 20-fold (3). TB has also been reported to exacerbate HIV infection (4). In South Africa, 76% of all TB cases registered in 2014 were HIV coinfected and on treatment with combination antiretroviral therapy (cART) (5). Acute HIV-1 infection is characterized by extremely high viremia, which the host immune system is subsequently able to partially control to a stable set point. Acute viremia is accompanied by a precipitous drop in tissue and circulating CD4^+^ T cells, a cytokine storm that never fully resolves, and generalized immune activation that has been associated with detrimental clinical outcome (6). Together, HIV and TB coinfection lead to rapid CD4^+^ T cell depletion (7), high viral load, and uncontrolled immune activation that exacerbates progression to end-stage AIDS. The large-scale application of cART has improved the quality of life of HIV-1-infected individuals and has reduced the incidence of active TB. Despite these public health gains, individuals on cART may still develop active TB at different stages of HIV infection (8), highlighting the need to better understand the underlying mechanisms of disease pathogenesis in coinfected individuals to facilitate better clinical management and disease prevention.

One of the more severe yet rare forms of TB is spinal TB, also known as Pott’s disease (9). In this condition, *M.tb* infiltrates *via* hematogenous spread from a pulmonary source (either active or latent) the spinal column where vertebrae, intervertebral disks and extradural tissue become infected with the formation of a “cold” abscess around the lesion (9). Recent reports have suggested that certain strains of *M.tb* exhibit enhanced affinity for osteoblasts (10). The vast majority of cases present with localized paraspinal infection without dissemination to the meninges (11). In extreme cases, paralysis may ensue as a result of spinal cord compression and loss of innervation. The disease normally resolves with anti-TB chemotherapy but surgical intervention involving wound debridement and insertion of a stabilizing bone graft (6) is necessary in severe cases (9). We previously described the cellular composition of spinal TB granulomas in HIV-infected patients, which resembled those of pulmonary TB granulomas in HIV-uninfected patients despite some subtle differences (12). In coinfected patients, spinal granulomas lacked organization and structure, CD4^+^ T-cell infiltration was restricted, and cellular composition was altered. In both HIV-infected and -uninfected individuals, immune cell populations included macrophages (CD68^+^), multinucleated giant cells, and CD3^+^, CD4^+^, and CD8^+^ T-cells. Nevertheless, little is known regarding the spectrum of immune cells involved and their exact contribution to TB immune responses in the spinal cord. The role of B-cells, plasma cells, epithelioid cells, and mesenchymal stem cells (MSCs), which have been characterized in pulmonary TB, have yet to be explored in spinal TB. Examination of the humoral arm of the anti-TB immune response has revealed that detection of anti-AG85 IgG antibodies is associated with favorable clinical outcomes in Mexican-American populations with pulmonary TB (13). Similarly, animal studies have demonstrated protective responses in mice inoculated with *M.tb*-specific IgG antibodies (14) and reduced disease severity in mice inoculated with *M.tb*-specific IgA antibodies (15).

In this study, we aimed to advance our understanding of the site-specific immune response within the discrete abscess of the spine by investigating the role of cell types such as B-cells, plasma cells, and MSCs in the immune response against TB. Furthermore, we sought to explore the possible implications of immune cell trafficking to the site of infection on the HIV immune response. Finally, we wanted to determine what, if any role the humoral immune response has at the site of spinal TB infection and how it may relate to the hypergammaglobulinemia associated with HIV infection.

## Materials and Methods

### Patient Study Cohort

Study participants were recruited from the Spinal Unit, King George V Hospital (now King Dinuzulu Hospital), Durban, South Africa between 2002 and 2003. This is a specialized referral within a public hospital that manages complex, often unresolved spinal pathologies including trauma and infections. Details of the unit and the cohort are provided elsewhere (12). Briefly, patients with clinical symptoms and radiological signs of spinal TB and who had not resolved their pathology despite anti-TB treatment for 5–24 weeks were recruited into the study. Patients with pulmonary TB, immunosuppressive disorders (other than HIV), and those on chronic steroids for clinical management of congenital disorders were excluded from the study. Radiological suspicion of spinal TB was confirmed with MRI scans, which also directed surgical debridement and graft insertion to the specific site of infection.

All participants in the study provided written informed consent. For children, consent was obtained from their parent(s) or guardian(s). The Biomedical Research Ethics Committee of the University of KwaZulu-Natal approved the study (H112/02).

### Sample Collection and Diagnostic Tests

To investigate the pathology and cellular architecture of granulomatous regions in the spine of patients, we used tissue fragments, excised during surgery from regions close to the vertebrae and proximate to the dura of the spinal cord, for histological analysis. The precise location of biopsies used to prepare the formalin-fixed paraffin-embedded (FFPEs) relative to the spinal cord and vertebrae was determined using X-ray images and MRI scans of the infected area. Abscess wall biopsies were collected from tissue proximate to the areas of bone destruction and vertebrae, usually anteriorly and close to the spinal cord. Biopsies were also harvested from abscesses surrounding the vertebrae with bone and disk destruction and involvement. A detailed description with related X-ray images is presented in Danaviah et al. (12).

Granulomatous tissue was collected during surgical removal of diseased bone, tissue, and the psoas abscess formed by the *M.tb* infection around the spinal column. Whole blood (EDTA anticoagulant) was also collected at surgery from each patient. Blood samples were submitted for T-cell quantification (CD4 and CD8 absolute and percentage counts) as well as for HIV serological diagnosis (Organon Teknika Vironostika and confirmatory Murex Wellcozyme HIV1+2 GAC assay). Plasma viral loads were performed on all samples that were serologically positive using the NucliSens™ QT kit, Organon-Teknika (Table 1).

**Table 1:** Clinical and demographic data of the patient cohort.

Parameter	HIV negative (*n* = 12)	HIV positive (*n* = 14)
Mean age ± SD (years)	35.8 ± 22.0	28.7 ± 15.7
Range	2.00–71.00	2.00–65.00
Gender			
Male (%)	4 (30.77)	5 (31.25)
Female (%)	9 (69.23)	11 (68.75)
Mean absolute CD4 count ± SD (cells/μl)	869.94 ± 549.48	544.24 ± 388.50
Range	319.00–2,254.20	74.47–1,080.50
Mean percentage CD4 count ± SD (%)	43.36 ± 15.97^a^	23.58 ± 10.27^a^
Range	24.86–72.00	8.82–43.00
Mean absolute CD8 count ± SD (cells/μl)	870.57 ± 710.53	1,098.09 ± 739.79
Range	194.00–1,269.00	26.99–2,512.00
Mean percentage CD8 counts ± SD (%)	37.46 ± 10.00^a^	57.39 ± 12.82^a^
Range	26.84–59.00	30.00–84.00
CD4:CD8 ratio	1.25 ± 0.56	3.63 ± 12.02
Range	0.55–2.23	0.10–45.39
Mean viral load ± SD (log copies/ml plasma)		4.57 ± 0.93
Range		2.92–6.39

*^a^Denotes the statistical significance*.

### Sample Processing

Tissue was placed in formalin (4% formaldehyde + 0.9% NaCl, 1:8 v/v) within 1 h of collection, and using conventional protocols, was processed and embedded in paraffin wax to produce (FFPE) blocks. Serial ultra-thin sections (5 µm thickness) were cut from each block for hematoxylin and eosin (H&E) staining (first section) and immune-localization studies (subsequent sections).

### Immune-Localization Studies

Specimens were selected based on H&E staining, with samples displaying intact granulomas chosen for the current study. Different cell subsets localized in the granuloma were identified using antibodies directed against standard cell-type specific markers (Table 2). The number of positively stained cells for a particular antibody in a selected region of interest (ROI) (at the same magnification) was quantified, manually, for all the samples by the same investigator (Debapriya Bhattacharya). The nuclei of stained and unstained cells were counted to determine the absolute number of cells per ROI.

**Table 2:** Antibodies used for identification of distinct immune cell types.

Antibodies	Cell types stained	Clone
CD3	T cells	Similar to F7.2.38
CD68	Monocyte/macrophage	PG-M1
Pax5	Pro B to mature B cells	DAK-Pax5
CD138	Plasma B cells	MI15
CD29	Mesenchymal stem cells	B3B11
p24	HIV protein	Kal-1

Immunocytochemistry was performed using the Envision TM Flex mini kit according to the manufacturer’s standard protocols. Briefly, the procedure involved de-paraffinization of sections with xylene, followed by hydration in graded ethanol and unblocking of antigen sites by incubation of sections in retrieval buffer at medium to high temperature in a microwave as per the manufacturer’s protocol (Envision TM Flex mini kit; Code K8023, Dako, Denmark). The peroxidase blocker of the kit was then used to block endogenous peroxidase activity, followed by incubation of sections with the primary antibody for 20 min. Next, samples were incubated with HRP-conjugated secondary antibodies for 40 min followed by visualization with the DAB (3,3′Diaminobenzidinetetrahydrochloride) chromogen and nuclear visualization using Mayer’s Hematoxylin (Biocare Medical, CA, USA).

For double staining of HIV protein p24 and CD68 or CD3, we used the Envision TM G/2 Double stain system. The procedure is similar to the single stain procedure up to incubation of retrieval buffer; after that, the sections were treated with dual endogenous enzyme blocker followed by incubation with the primary p24 antibody for 30 min. The section was then incubated with the secondary antibody anti-mouse or anti-rabbit IgG for 30 min followed by incubation with polymer HRP. Sections were then washed and incubated for color development using DAB solution. Subsequently, sections were re-incubated in double stain blocker followed by incubation with second primary antibody (either CD68 or CD3) and then incubated with linker followed by Polymer AP and colored by permanent red working solution (Envision double staining kit, Dako). The sections were then washed with distilled water followed by hematoxylin staining of the nucleus.

### Immunoglobulin Quantification

Immunoglobulin (Ig) M, total IgG, and the isotypes IgG1, IgG2, IgG3, and IgG4 from the serum of patients were assayed by a Luminex microbead-based multiplexed assay using commercially available kits according to the manufacturer’s instructions (Bioplexpro™ Human isotyping panel, 6 Plex, Bio-Rad).

### Statistical Analysis

Data were analyzed in Stata V11 and SPSS. Given the small sample size of our cohort, we used non-parametric tests as well as Tukey’s one-way ANOVA of significance and correlation where a 5% level of significance was accepted as demonstrating differences and associations, respectively. Graphs illustrating these relationships were also generated in Stata V11.

## Results

### Clinical and Demographic Data of the Patient Cohort

Previously, it has been demonstrated that various immunocytes infiltrate within spinal granulomas of HIV-1-uninfected and -infected TB patients (12), but the role of these cells in disease pathogenesis remains largely unknown. Furthermore, we hypothesized that MSCs may play a role in Pott’s disease, as these cells were recently shown to contribute to suppressed immune responses in a mouse model of pulmonary TB (16). Based on availability of FFPE blocks, we selected 30 patients, including 12 HIV-uninfected and 14 HIV-infected individuals with a mean age of 35.8 ± 22.0 and 28.7 ± 15.7 years, respectively (Table 1). As previously reported, most of the participants in our cohort were females (Table 1). Absolute CD4 counts in HIV-infected patients were not significantly different to those of HIV-uninfected patients. However, the percentage of CD4 cells was significantly decreased in HIV-positive patients (Table 1; *p* < 0.01). In contrast, while the absolute CD8^+^ counts where similar between the two groups, the percentage of CD8 cells was significantly higher in HIV-positive patients (Table 1; *p* < 0.01). Viral loads in the HIV-infected group were high (average = 4.57 ± 0.93 log_10_ copies/ml; range = 2.92–6.39 log_10_ copies/ml) and none of the patients presented with viral suppression since they were not on cART at the time of sample collection.

### Architecture and Immune Cell Content of Granulomas

**Figure 1:**
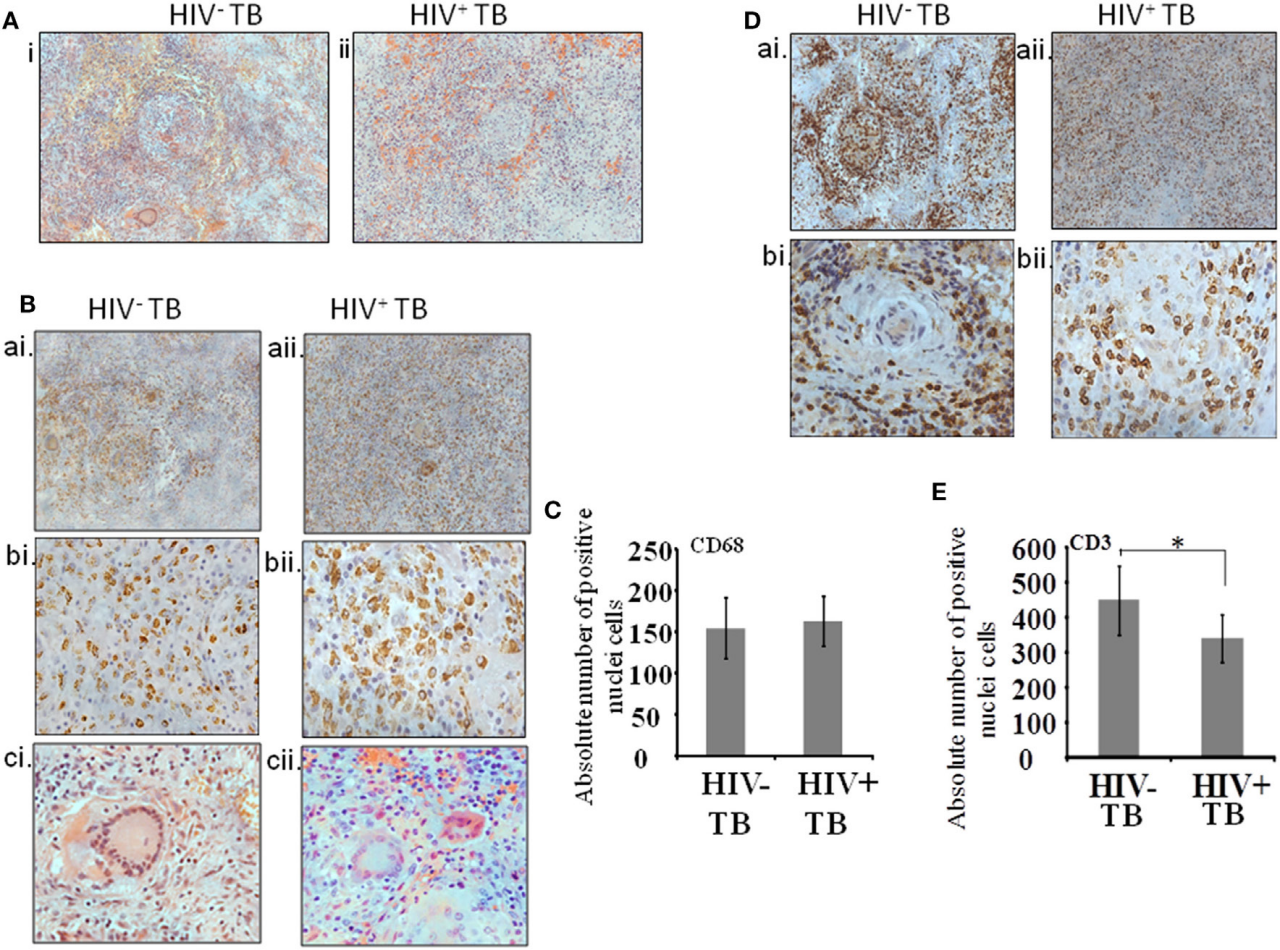
Cellular architecture of spinal tuberculosis (TB) granulomas. [**(A)**, i,ii] Images show a representative area of a granuloma stained with H&E. **(B)** Distribution of CD68^+^ cells within spinal TB granulomas of HIV^−^ and HIV^+^ patients. [**(B)**, ai,aii]: 20× and [**(B)**, bi,bii]: 40× microscopic view of anti-CD68 stained cells; [**(B)**, ci,cii]: hematoxylin and eosine stained giant cells. **(C)** Pooled absolute numbers of CD68^+^ cells in different specimens of HIV^+^ and HIV^−^ samples. **(D)** Distribution of CD3^+^ cells. [**(D)**, ai,aii]: 20× and [**(D)**, bi,bii]: 40× microscopic view of anti-CD3 stained cells; **(E)** pooled absolute numbers of CD3^+^ cells in different samples (*denotes the statistical significance).

Serial sections were assayed as follows. The first section was stained with H&E and the remaining sections were used to localize the cells stained by antibodies against CD68, CD3, CD138, CD29, Pax5, CD68 with p24, and CD3 with p24 (Table 2). In order to analyze immune-localization of various cell types in the two groups, we manually counted cell numbers within regions of interest. Histologic examination of low-power images of H&E-stained tissue revealed the classic structure of a typical granuloma in the HIV-uninfected specimens (Figure 1A, i). However, a more diffuse or heterogeneous structure was typical of HIV-infected tissue (Figure 1A, ii). Both groups displayed the presence of macrophages and T-cells in H&E-stained sections as well as in immune-localized stained tissue (Figures 1A,B,D). We noted the presence of CD68^+^ monocytes and multinucleated giant cells in both HIV-uninfected (Figure 1B, ai,bi,ci) and -infected tissues (Figure 1B, aii,bii,cii). The distribution of macrophages was more contained within the granulomas in the HIV-uninfected samples, but diffuse in the HIV-infected samples. Multinucleated giant cells displayed the same morphology in both groups (Figure 1B, ci,cii) and absolute numbers of CD68^+^ cells were not significantly different between HIV-infected and -uninfected tissue (*p* = 0.36) (Figure 1C). Similarly, T-cell distribution as demonstrated by anti-CD3 antibody staining was confined largely to the periphery with specific staining within the granuloma in HIV-uninfected specimens (Figure 1D, ai,bi). HIV-infected tissue again displayed diffuse staining where CD3^+^ T cells were scattered throughout the granulomatous region (Figure 1D, aii,bii). We also noted that total numbers of CD3^+^ cells were higher in HIV-uninfected compared with HIV-infected specimens (*p* = 0.03) (Figure 1E).

**Figure 2:**
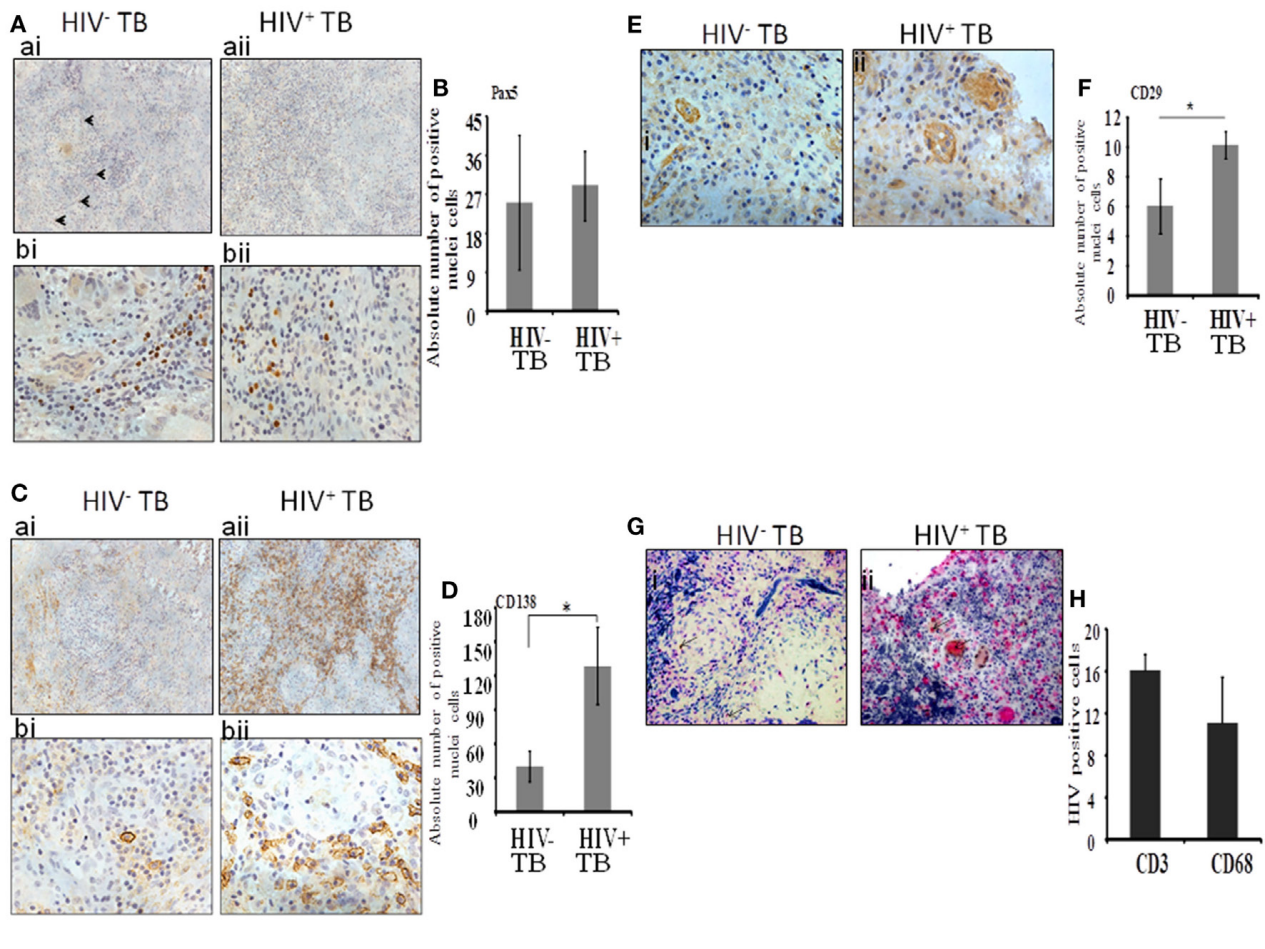
Plasma B cells and mesenchymal stem cells are increased dramatically during HIV-tuberculosis (TB) coinfection. **(A)** Distribution and percentage of Pax5^+^ cells within spinal TB granulomas. [**(A)**, ai,aii]: 20× and [**(A)**, bi,bii]: 40× microscopic view of Pax5-stained cells; **(B)** pooled absolute number of Pax5^+^ cells in different samples. **(C)** Distribution and percentage of CD138^+^ plasma-B cells within spinal TB granulomas. [**(C)**, ai,aii]: 20× and [**(C)**, bi,bii]: 40× microscopic view of CD138^+^ stained cells; **(D)** Pooled absolute numbers of CD138^+^ cells in different samples. **(E)** Percentage of CD29^+^ cells within spinal TB granulomas of HIV-negative and -positive patients. [**(E)**, i,ii]: 40× microscopic view of anti-CD3 stained cells; **(F)** pooled absolute number of CD29^+^ cells in different samples. **(G)** CD4^+^ T cells and CD68^+^ macrophages are a reservoir of HIV. [**(G)**, i,ii]: 40× microscopic view of CD3^+^ or CD68^+^ cells double-stained for localization of HIV protein p24; pink is stained for either CD3^+^ or CD68^+^ cells, brown is for p24 protein of HIV, and brownish pink color is for double staining. **(H)** Pooled absolute number of double-stained cells in different samples (*denotes the statistical significance).

Next, we analyzed immature B cells and plasma cells within the spinal granulomas. We first used an antibody to Pax5, a master transcription factor of the B cell lineage expressed in precursor B cells up to mature B cells. In both HIV-uninfected and coinfected tissue, we noted the presence of Pax5-positive B cells diffusely distributed within the tissues, but not within the center of granulomas (Figure 2A). We extended our analysis by including anti-CD138 antibodies, which stain terminal plasma cells. A notable difference in the level of staining was observed with the HIV-infected specimens showing diffuse positive staining over large portions of tissue (Figure 2C, aii,bii), whereas in contrast, HIV-uninfected tissue displayed limited staining (Figure 2C, ai,bi). In both groups, plasma cells were observed at the periphery of granulomas. When we enumerated the absolute numbers of Pax5^+^ and CD138^+^ cells, we found a significant increase in CD138^+^ plasma cells (Figure 2D; *p* = 0.01) in HIV-infected compared with -uninfected tissue, whereas no difference was observed in the numbers of Pax5^+^ B cells (Figure 2B; *p* = 0.49).

Mesenchymal stem cells were identified by immunolocalization of the CD29 Ab as per our previous work (16) whereas with the current study, only one marker was used to identify this immune cell subset. These cells were present in both HIV-negative and -positive samples (Figure 2E, i,ii). In HIV-uninfected samples, MSCs were located in the peripheral of granulomas whereas in HIV-infected samples, MSCs were more sparsely distributed and were dispersed throughout the granulomas. Interestingly, significantly increased numbers of CD29^+^ MSCs were present in the HIV coinfected compared with the HIV-uninfected group (*p* = 0.003) (Figure 2F).

Finally, to investigate the distribution of HIV-infected macrophages and T-cells, we performed dual staining of anti-p24 HIV antibody with the T-cell-specific anti-CD3 antibody or with the macrophage-specific anti-CD68 antibody. Both CD3^+^ T cells and CD68^+^ cells displayed colocalization with HIV-1 anti-p24 antibody staining (Figure 2G, i,ii and Figure 2H).

### HIV Infection Induces IgG and/or IgM Responses

Since CD138^+^ plasma cells are significantly increased in HIV-positive patients, we explored the dynamics of their activation and their specificity. For this purpose, we measured a surrogate marker of activation, total serum immunoglobulin level, since IgGs are mainly antigen-specific and T-cell-dependent whereas IgMs are mainly T-cell independent.

**Figure 3:**
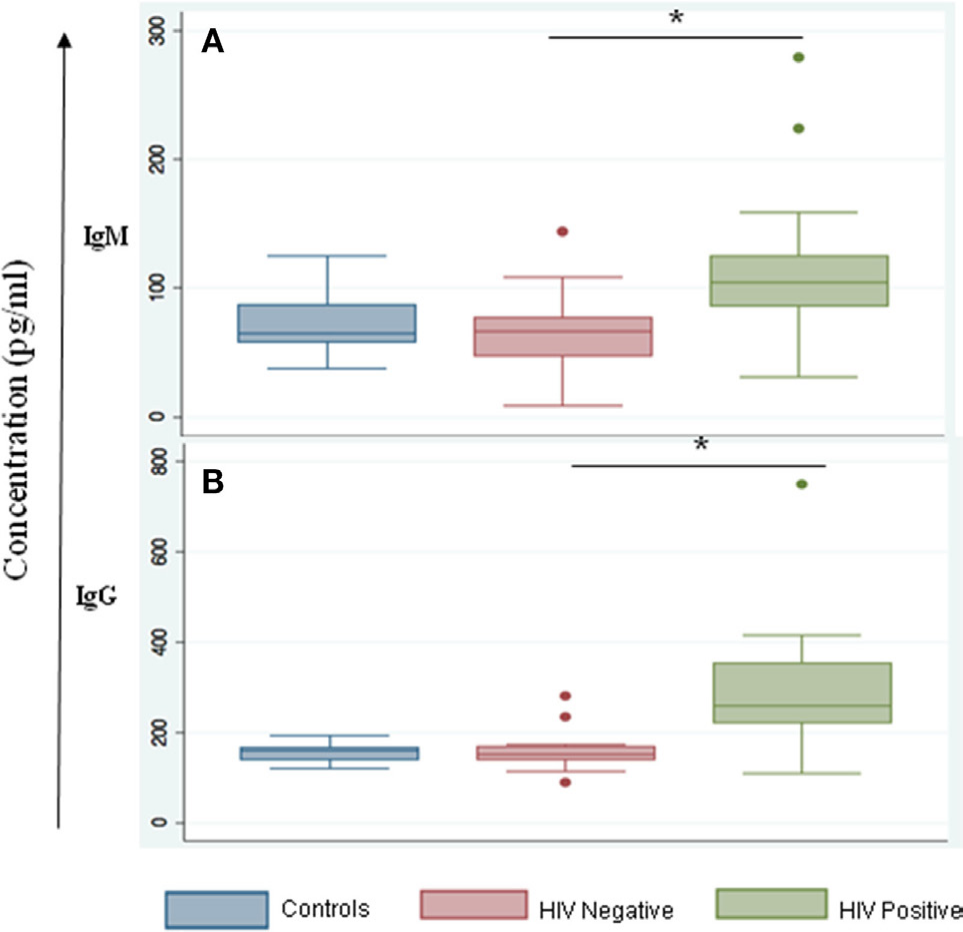
IgM and IgG serum levels are altered during HIV-tuberculosis (TB) coinfection. Graphical representation of immunoglobulin expression levels measured in control samples that were HIV- and TB-negative (blue); spinal TB-positive but HIV-negative (salmon); spinal TB-positive and HIV-positive (green). **(A)** Total serum IgM. **(B)** Total serum IgG (*denotes the statistical significance).

**Figure 4:**
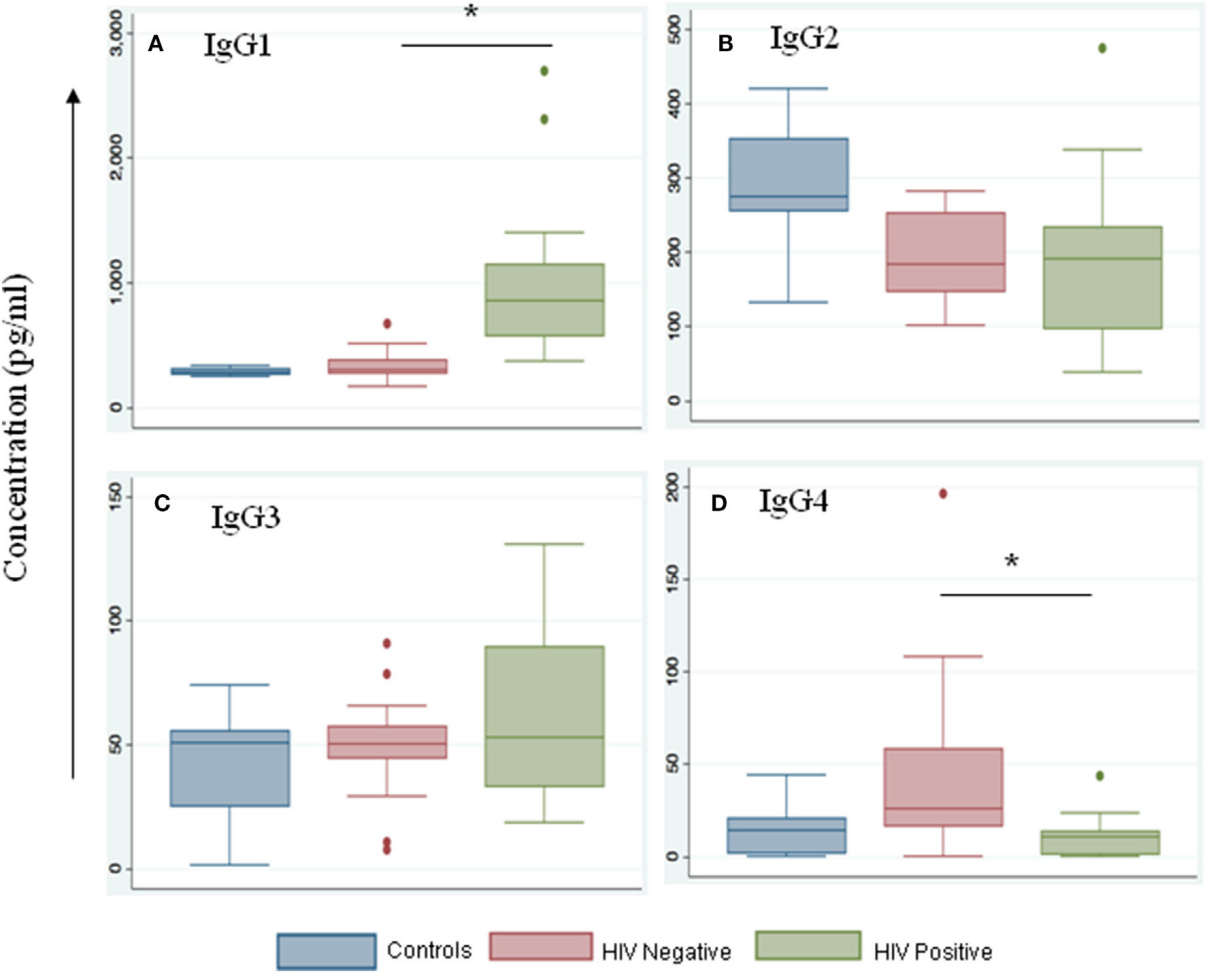
IgG1 levels are increased during HIV-tuberculosis (TB) coinfection. Representative box-plots illustrating expression levels of IgG subclasses IgG1 **(A)**, IgG2 **(B)**, IgG3 **(C)**, and IgG4 **(D)** in control (blue), HIV-negative (salmon), and HIV-positive (green) samples. Control samples are HIV- and TB-negative (*denotes the statistical significance).

We noted that both IgG and IgM levels were significantly increased in plasma samples of the HIV-positive group (*p* = 0.003 and 0.01, respectively, by Tukey’s one-way ANOVA) (Figures 3A,B). In the HIV-negative group, plasma IgG and IgM concentrations were low and were similar to concentrations measured in the control samples (HIV-negative and *M.tb*-negative plasma samples) (Figures 3A,B). This suggests that the increased infiltration of plasma cells in HIV-positive tissue samples might contribute to increased activation of the humoral immune response in coinfected individuals. Finally, we determined IgG subtypes (Figure 4), which revealed that IgG1 was significantly increased in HIV-positive samples (*p* = 0.0001) whereas IgG4 was significantly increased in HIV-negative samples (*p* = 0.047) (Figures 4A,D).

## Discussion

The host and pathogen in TB and HIV infection are in a fine balance that is influenced by a number of inter-related factors (17). Primary among them is the host immune response. In this study, we present evidence for involvement of the humoral and cellular arms of the immune response in spinal TB and that HIV coinfection significantly enhances B-cell trafficking to spinal TB granulomas. We further present the novel finding that coinfection augments MSC localization to the site of infection in granulomas. Similarly, total serum IgG and IgM levels were increased in coinfected patients, and IgG subclasses were differentially affected by the HIV status of the host.

In our previous study, we focused on the distribution and density of macrophages and T cells, mainly CD4 and CD8 cell populations, and compared them in spinal TB granuloma of HIV-infected or uninfected patients (12). However, in the present study, we expanded these findings to focus on B cells, plasma cells, and MSCs as well as investigating the humoral response by measuring IgM and IgG levels. For completeness and consistency, we again stained macrophages and CD3-positive T cells such that we now gain an understanding of the relationship between and the activation status of T-cells, B-cells, and plasma cells in spinal TB of HIV-infected or uninfected patients. Also, we found that anti-HIV p24 antibody is present in T-cells and macrophages within the granulomas. These findings support our hypothesis that TB may enhance HIV replication within T-cells and macrophages inside granulomas (18), which is consistent with the findings of other studies (4, 19–21).

While much is known about the role of T-cells in the host immune response against TB, the role of B-cells and the antibodies they produce remain incompletely understood. A few studies have provided evidence for a protective role of antibodies in Mtb infection (22, 23). Further, B-cell localization studies using anti-CD20 antibody in a primate model identified positive cells in aggregates as well as scattered throughout the lymphocytic cuff of the granulomas (24). In our study, we found a peripheral distribution of Pax5-positive B-cells in HIV-negative TB tissue and a more diffuse distribution in HIV-positive TB-infected tissue. The difference in distribution may be attributed to the host (primate versus human). Severely suppressed B-cell populations have previously been identified in human peripheral blood samples from pulmonary TB patients as compared with healthy controls (25). The breadth of the antigen-specific B-cell response in TB patients is limited but the inflammatory response, as evidenced by upregulation of inflammatory cytokines, is robust (25). Our findings revealed limited numbers of B-cells in infected tissues, which was not dependent on HIV status. Whether these responses are antigen specific cannot be gleaned from our data and will require further investigation.

We found that plasma cells were substantially increased in HIV-infected specimens, suggesting localized B-cell maturation. Similarly, high numbers of plasma cells were observed at the site of infection for pulmonary TB in non-human primates (24). The disparity between plasma cell (increased) and total B-cell (decreased) numbers that we observed in HIV-infected TB tissues was also found in the bone marrow of HIV-infected individuals (26). To explore consequences of increased plasma cells, we measured antibody responses and found increased total IgM and IgG, especially IgG1, in HIV-positive TB patient serum. Serum IgM and IgG levels in HIV-negative TB samples were very similar to those of healthy individuals. These findings are consistent with the notion that T helper responses during chronic HIV infection are skewed toward Th2 type immunity (27). This also supports the view that HIV-1 Env-specific antibody responses are dominated by IgG1 (27). HIV-induced Th2 skewing of the immune response may contribute to its effects on TB coinfection, as protective immunity to TB requires Th1 responses (28). In contrast to our study, others report that IgG-antigen-secreting cells are significantly elevated in acutely infected individuals but that these cells do not impact Ab production (29–33). Additional studies have shown that elevations in plasma IgG levels during chronic HIV infection are antigen non-specific, resulting in partial hypergammaglobulinemia (34). More recently, it was reported that B cell dysfunction impacts antibody-producing cells in the intestinal mucosa where cells of HIV-1-infected individuals display decreased capacity to switch from the production of IgM to IgA or IgG antibodies (35). B-cell dysfunction is a hallmark of HIV infection and the disparity in B cell frequencies that we observed may, therefore, be attributed, in part, to HIV coinfection. Increased levels of IgG4 subtype in HIV-uninfected TB-positive patients may also indicate the presence of lymphadenopathy as a result of TB-infection alone.

We found MSCs in both HIV-infected and -uninfected specimens. These progenitor cells are able to differentiate into distinct cell types such as osteoblasts and have been used in cellular therapeutics from aiding in tissue graft acceptance to adjuvants in TB infections (36). We have shown the presence of osteoblasts in HIV-infected patients in our cohort (12), which may have been derived from MSCs. We speculate that the presence of MSCs in bone tissue may benefit patients during post-surgery recovery, formation of new bone, and receipt of tissue allografts, regardless of HIV status (37). Given that MSCs also reduce inflammation and prevent tissue damage (38), these cells likely protect against exacerbated tissue and bone degeneration that may lead to paralysis in the large majority of HIV/TB coinfected patients.

The TB vaccine BCG has been in use for almost a century but provides only limited to no protective immunity against adult pulmonary disease. Understanding the humoral arm of the TB immune response is likely to contribute new knowledge on the protective measures required against TB infection as was highlighted in a recent NIAID-convened discussion on Mtb immune evasion and vaccine development (39). Understanding the expansion of the innate and adaptive immune responses over the course of disease progression remains a bottleneck to developing suitable vaccines. While the full gamut of the Ab-mediated response may not be fully activated or effective during TB or TB/HIV infection, it is undoubtedly a key aspect of disease containment of intra-cellular pathogens such as TB (40). Our findings add to the substantial body of work that describes the immune response to TB and HIV but provides more insight into the innate mechanisms of the immune response. In addition, we provide a possible impact of HIV/TB coinfection on the phenomenon of hypergammaglobulinemia in spinal TB. Our data suggest that B-cell dysfunction is a hallmark of HIV infection in TB coinfected patients, as is hypergammaglobulinemia.

## Conclusion

The intimate association between HIV and TB necessitates efforts to understand the humoral and cellular arms of the immune response in coinfected hosts. The spinal TB granuloma provides a unique and isolated, yet highly reactive site of coinfection. Our studies demonstrate that the dynamics of immune cell trafficking and function greatly differ between HIV-infected and -uninfected hosts. We provide novel findings regarding B-cells, plasma cells, and MSCs at the site of spinal TB infection and show that HIV coinfection perturbs plasma cell and MSC trafficking. Additional studies are needed to fully dissect humoral immune responses in this coinfection model, which should be informative for the development of novel protective measures against TB and TB/HIV coinfection.

## Ethics Statement

All participants in the study provided written informed consent. For children, consent was obtained from their parent(s) or guardian(s). The Biomedical Research Ethics Committee of the University of KwaZulu-Natal approved the study (H112/02).

## Author Contributions

DB and SD designed the experiment did the work, analyzed it, and wrote the paper, DM and NA helped in Luminex microbead-based multiplexed assay, PM analyzed the data and wrote the paper, TN and GD designed and supervised the experiments, analyzed the data, and wrote the paper.

## Conflict of Interest Statement

The authors declare that the research was conducted in the absence of any commercial or financial relationships that could be construed as a potential conflict of interest.
